# Multi-channel electronic and vibrational dynamics in polyatomic resonant high-order harmonic generation

**DOI:** 10.1038/ncomms6952

**Published:** 2015-01-22

**Authors:** A. Ferré, A. E. Boguslavskiy, M. Dagan, V. Blanchet, B. D. Bruner, F. Burgy, A. Camper, D. Descamps, B. Fabre, N. Fedorov, J. Gaudin, G. Geoffroy, J. Mikosch, S. Patchkovskii, S. Petit, T. Ruchon, H. Soifer, D. Staedter, I. Wilkinson, A. Stolow, N. Dudovich, Y. Mairesse

**Affiliations:** 1 Université de Bordeaux—CNRS—CEA, CELIA, UMR5107, F33405 Talence, France; 2 National Research Council Canada, 100 Sussex Drive, Ottawa, K1A 0R6 Ontario, Canada; 3 Department of Physics, University of Ottawa, 150 Louis Pasteur, Ottawa, K1N 6N5 Ontario, Canada; 4 Department of Physics of Complex Systems, Weizmann Institute of Science, Rehovot 76100, Israel; 5 CEA, IRAMIS, Lasers, Interactions and Dynamics Laboratory—LIDyL, CEA-SACLAY, F-91191 Gif-sur-Yvette, France; 6 Max-Born-Institute, Max-Born-Strasse 2A, 12489 Berlin, Germany; 7 Université de Toulouse—CNRS, LCAR-IRSAMC, 31062 Toulouse, France; 8 Department of Chemistry, University of Ottawa, 10 Marie Curie, Ottawa K1N 6N5 Ontario, Canada K1N 6N5

## Abstract

High-order harmonic generation in polyatomic molecules generally involves multiple channels of ionization. Their relative contribution can be strongly influenced by the presence of resonances, whose assignment remains a major challenge for high-harmonic spectroscopy. Here we present a multi-modal approach for the investigation of unaligned polyatomic molecules, using SF_6_ as an example. We combine methods from extreme-ultraviolet spectroscopy, above-threshold ionization and attosecond metrology. Fragment-resolved above-threshold ionization measurements reveal that strong-field ionization opens at least three channels. A shape resonance in one of them is found to dominate the signal in the 20–26 eV range. This resonance induces a phase jump in the harmonic emission, a switch in the polarization state and different dynamical responses to molecular vibrations. This study demonstrates a method for extending high-harmonic spectroscopy to polyatomic molecules, where complex attosecond dynamics are expected.

## Abstract (web summary)

Strong-field ionization in molecules is more complex than in atoms with multiple channel dynamics and a coherent superposition of electronic states. Here, Ferré *et al*. develop multi-modal spectroscopy to measure those dynamics and reveal a resonant to non-resonant high-harmonic generation transition.

## Graphical abstract

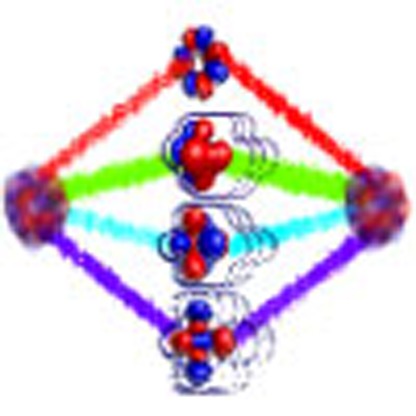

In the simplest view of molecular strong-field ionization, the most probable valence electron to be removed by a strong laser field is the most weakly bound one, namely that from the highest occupied molecular orbital. This assumption is based on quasi-static, single active electron pictures and the exponential decay of the tunnel ionization probability through a field-lowered Coulomb barrier^1^. However, even in a quasi-static picture, the tunnel ionization probability also depends on the spatial structure of the molecular orbital^2^,^3^. Furthermore, more than one electron could get driven coherently by the laser field. The timescales of electron motions in molecules may not be fast compared with the laser period and the adiabatic approximation inherent to the quasi-static picture may fail: non-adiabatic multi-electron dynamics can ensue^4^. Consequently, strong-field ionization is significantly more complex for molecules than for atoms, with possible contributions from multiple channels, leaving the ion in a coherent superposition of electronic states. Such ionic excitation can result in multi-electron processes and ultra-fast charge migrations on an attosecond timescale^5^,^6^.

The signatures of multi-channel ionization in above-threshold ionization (ATI)^7^ and high-harmonic generation (HHG)^8^,^9^,^10^ are fundamentally different. The ATI process for each ionization channel can be understood as a coherent superposition of continuum photoelectron wavepackets, which are created during every half cycle of the laser field. However, the ionization pathways leading to different ionization continua are distinguishable and will hence simply add up incoherently. ATI is a single-channel interferometer, different ionization channels produce independent ATI progressions in the photoelectron kinetic energy spectrum. In contrast, in HHG, the different ionization channels connect the same initial and final states (that is, neutral ground electronic state) and therefore their contributions are coherently summed in the harmonic emission. The HHG mechanism can thus be described as a multi-channel interferometer on the timescale of half a laser cycle. The weights of the interfering ionization channels are mainly determined by the ionization yields and the recombination dipole moments^11^,^12^.

In many molecular systems, the recombination process can involve resonant transitions due to the presence of autoionizing states or shape resonances. In that case, the recolliding electron can get trapped in the vicinity of the parent ion for typically several hundreds of attosecond before recombining to the ground state^13^,^14^. On the contrary to autoionization, shape resonances have an energy bandwidth—typically a few eV—that depends strongly on the non-spherical and non-local characters of the ionic potential. Consequently, their energy and lifetime can vary as function of the molecular geometry. These scattering patterns related to the molecular field enhance the photoabsorption cross section and are imprinted in the photoelectron asymmetry parameter. In HHG, the basic properties of the harmonic—their amplitude, phases and polarization state—may be dramatically influenced by such peculiar resonant electron scattering. Their presence may significantly increase the contribution of inner ionization channels, commonly hidden in HHG experiments. Despite their importance in molecular HHG, very few experiments have reported signatures of resonances. A maximum in the harmonic spectrum from xenon atoms has been attributed to a giant resonance^15^, but other experiments have shown that the shape of the harmonic emission in xenon critically depended on focusing conditions, such that maxima could appear or disappear in the spectrum^16^. Clearly, the identification of the resonances’ contribution requires the measurement of additional experimental parameters. Following this approach, Haessler *et al*.^17^ reported the measurement of a phase jump across a narrow resonance in ablation plasma plumes. An alternative is to add control parameters to the interaction and measure the response of the system to these parameters. For instance, molecular alignment is broadly used in HHG experiments for channel identification in diatomic and linear triatomic molecules^5^,^6^,^18^,^19^,^20^. It has allowed to interpret a maximum in the intensity from aligned N_2_ as a signature of a shape resonance in the lowest ionization channel^21^. However, for polyatomic molecules, field-free three-dimensional alignment remains challenging in general^22^. Therefore, we need a more direct control of the interaction.

In this letter, we combine complementary strong-field measurements to develop a multi-modal high-harmonic spectroscopy. To illustrate this, we chose a polyatomic system in which many channels can contribute to the harmonic emission: SF_6_, where the four lowest states of the cation (each multiply degenerate) lie within less than 3 eV as illustrated in Fig. 1. Each cation state of SF_6_ can be described in a first approximation by a single-hole configuration of the neutral molecule (Dyson orbital). Therefore, each subshell ionization channel corresponds to one cation state only and we will label the channels via their associated cation state. Due to its symmetry (O_*h*_ point group), this system cannot be spatially laser aligned in the laboratory frame. The identification of the channels contributing to HHG and the measurement of multi-channel ionization dynamics is thus particularly challenging. To discern the ionization mechanism, which is the first step involved in the harmonic emission, we performed fragment-resolved ATI measurements^23^ on SF_6_ for laser wavelengths ranging from 800 to 400 nm, revealing that multiple channels contribute to the strong-field ionization. Using high-harmonic spectroscopy, we performed a complete characterization of the emission—spectrum, spectral phase, polarization state—and used the laser ellipticity and vibrational excitation as control parameters. This approach reveals a clear transition between resonant and non-resonant HHG around 26 eV. Vibrational dynamics in the ground electronic state of SF_6_ (via stimulated Raman pumping) is a molecular control parameters that allows to explore the dependency of the resonance on the molecular geometry. The results of these combined experiments, corroborated by state-of-the-art *ab initio* calculations, allowed us to identify the inner ionization channels as the dominant contributors to HHG in SF_6_ and the prominent role of shape resonances in this mechanism.

## Results

**Figure 1:**
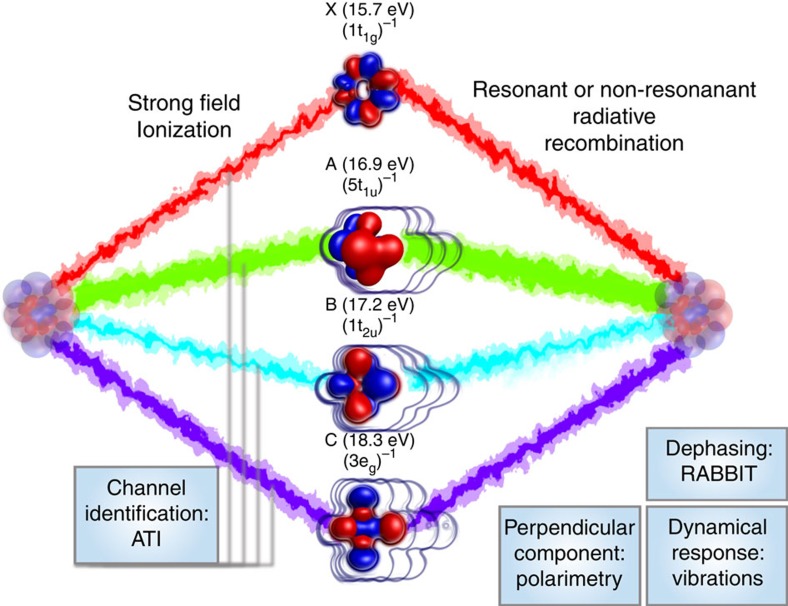
Schematic view of multi-channel HHG. Multiple open channels in strong-field ionization are identified with fragment-resolved ATI measurements. The field-free, vertical ionization potential of each channel is indicated next to the schematic view of the associated valence subshell of ionization. The radiative recombination process is modified by the presence of resonances, increasing some of the channels’ contribution to the harmonic emission.

Figure 1 shows the different ionization channels connecting the initial and final states, as well as their energies and symmetries. This figure also summarizes the different experiments described in the following.

### Strong-field ionization channels in SF_6_

ATI and HHG are closely related subcycle ionization phenomena. In ATI, strong-field ionization (SFI) releases electrons into the continuum around every maximum of the laser electric field. The emission is repeated every optical cycle throughout the pulse. As a result, the photoelectron spectrum shows discrete peaks separated by the photon energy *ℏω* of the driving laser. The absolute position of the peaks of this ATI comb is specific to each ionization channel:


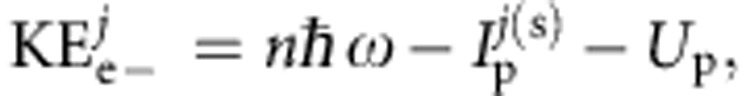


as it is determined by the Stark shifted (*s*) ionization potential 
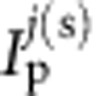
 of ionization channel *j* and the channel-independent ponderomotive shift *U*_p_ (*n* is an integer). At a constant intensity, the ATI combs of different channels give a direct measurement of their relative ionization potentials modulo the photon energy. In polyatomic molecules, one can exploit field-free fragmentation to better identify the different SFI channels. Using covariance or coincidence detection, the ATI spectra associated with different fragments are measured. How well different channels can be separated depends on the details of the unimolecular fragmentation process. In cases where specific fragments or a parent ion single out individual ionization continua, fragment-resolved ATI measurements become channel-resolved (the CRATI technique)^23^,^24^.

The position of an ATI comb in the photoelectron spectrum reflects the channel 
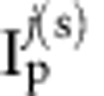
 modulo the photon energy. In general, if the energy difference between two ionization channels matches the photon energy of the driving laser, the ATI combs will overlap. For SFI of SF_6_, the situation is complicated by the fact that the states involved are significantly Stark shifted in the laser field. Moreover, these Stark shifts depend sensitively on the wavelength. We have estimated the ionization channel-specific dynamic Stark shifts computationally (see Supplementary Fig. 1). Importantly, if there is a strong resonance for a specific ionization channel, the Stark shifts might also diverge. As seen from Supplementary Fig. 1, there are channel-specific divergences for photon energies around 1.2, 1.4 and 2.7 eV, which corresponds to the energetic separation of the field-free X and A/B, A/B and C and X and C states of the SF_6_ cation. For photon energies in the vicinity of such a Stark shift divergence, the respective comb might be washed out entirely due to averaging over the laser bandwidth.

**Figure 2:**
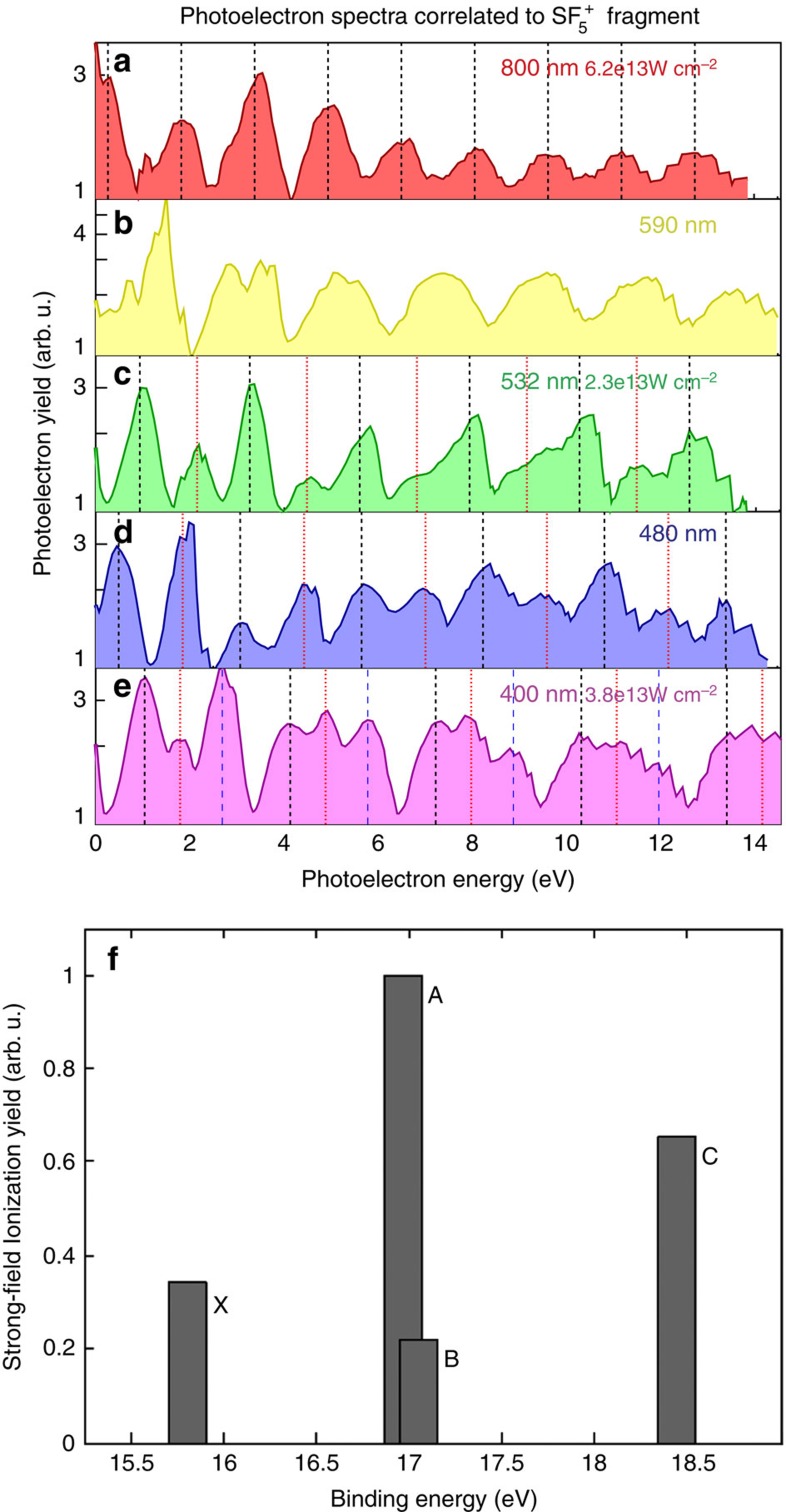
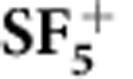
 fragment photoelectron spectra from SFI of SF_6_, at different laser wavelengths and intensities (note that the ground state of 
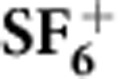
 is unstable). While a single ATI comb is observed for 800 nm (**a**), it splits up into two (**b**,**d**) and ultimately three (**e**) progressions for shorter wavelengths. The different ATI combs are marked by the sets of vertical lines, which are spaced by the respective photon energy. It can hence be concluded that multiple ionization continua with similar yields must be present in the SFI of SF_6_. For 800 nm, the multi-channel nature is obscured by the cation electronic states having energy level spacings, which happen to be multiples of the photon energy. (**f**) Calculated ionization yield for different channels at 800 nm. The X, A, B and C states are the ground and first three electronically (doublet) excited states of the cation.

To resolve different SFI channels, we varied the laser wavelength between 400 and 800 nm and performed covariance measurements, producing photoelectron spectra associated with the various fragments of SF_6_ (see Methods). In Fig. 2, we show spectra for the most abundant fragment 
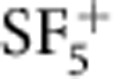
 (note that the ground state of 
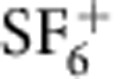
 is unstable). In the 800-nm photoelectron spectrum displayed in Fig. 2a, a single ATI comb is observed. However, if the photon energy is increased, the single comb starts to split (Fig. 2b–d), and at 400 nm three progressions of peaks, each spaced by the photon energy, are clearly visible (Fig. 2e). This demonstrates that at least three ionization continua contribute to the SFI of SF_6_ and that their yields must be comparable. For the 800-nm driving field, the multi-channel nature of the strong-field ionization process is obscured by the Stark shifted cation electronic states having ionization potentials congruent modulo the photon energy. The variation of the driving field frequency was required to resolve the multiple SFI channels involved.

An assignment of the ionization channels in Fig. 2 would require more reliable Stark shift calculations and a precise intensity calibration, since Stark shifts are in the eV range. This is beyond the scope of the present work. To quantify the relative yields of the active SFI channels, we would require knowledge of the branching ratios for ionic fragmentation from each of the populated cation states, which are unknown, along with a detailed analysis of the photoelectron spectra associated with all ionic fragments. Instead we present computationally determined channel-specific SFI yields for 800 nm by calculating the tunnel ionization probability 
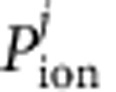
 for each channel *j* by using a previously described approach^25^,^26^ and averaging over all molecular orientations. Ionization calculations used a sin^2^ pulse matching the peak of a 1/2-cycle 800-nm pulse at 10^14^ W cm^−2^ peak intensity. The resulting yields are shown in Fig. 2f. Three main factors determine the channel SFI rates: (i) the ionization potential of the channel—the ionization rate decreases exponentially with *I*_p_; (ii) the topography of the ionizing molecular orbital—nodes in the plane perpendicular to the laser polarization tend to suppress the ionization probability; (iii) interchannel coupling, due to both the laser-induced dipole transitions and electron correlation—subcycle electronic transitions within the ion core can redistribute the final ionization amplitude between coupled channels. The first effect was long considered to be dominant: SFI mainly occurs from the highest occupied molecular orbital, even though deeper channels can be observed under specific conditions, such as in aligned molecules. However, in a CRATI study of *n*-butane^23^, it was shown that excited-state channels dominate over the ground-state channel, despite their higher *I*_p_s. The results from Fig. 2f are alike due to geometrical effects and interchannel coupling in SFI of SF_6_ which overcome the exponential dependence on *I*_p_, even for unaligned targets.

### High-harmonic spectrum

**Figure 3:**
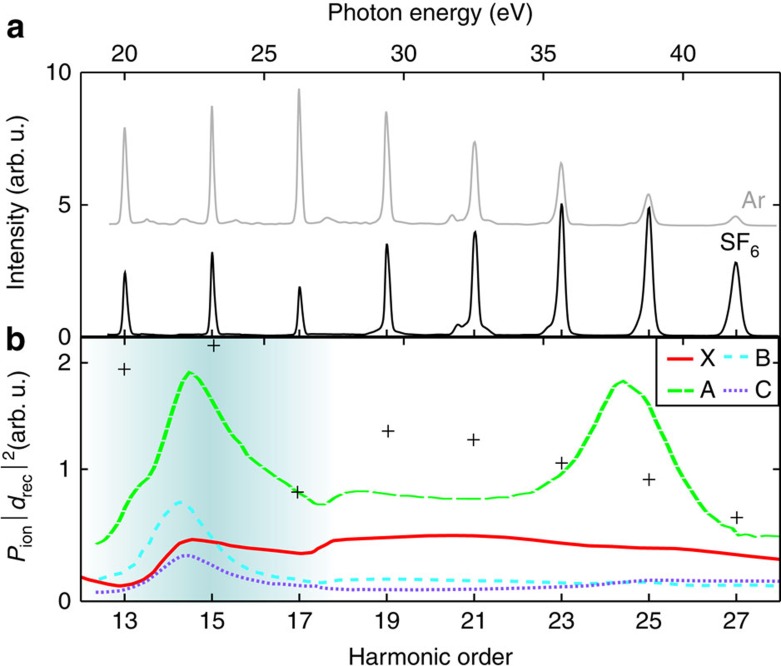
HHG from SF_6_. (**a**) Harmonic spectra generated in Ar (grey) and SF_6_ (black) at *I*≈1.3 × 10^14^ W cm^−2^. The argon spectrum is vertically shifted to improve visibility. (**b**) Harmonic spectra obtained after normalizing out the recolliding electron wavepacket. Experiment (crosses) and contribution from different channels.

SFI involving multiple channels generates multiple interfering pathways in HHG. The harmonic signal is determined by the coherent sum over all possible channels (see Fig. 1). Figure 3a shows an experimental harmonic spectrum generated in SF_6_ using 800 nm pulses at *I*≈1.3 × 10^14^ W cm^−2^. To distinguish the simple, single-orbital non-resonant picture from the multi-channel case, we compared our measurement with argon, an atomic system with a similar ionization potential to the first I_p_ of SF_6_. The SF_6_ spectrum extends to higher orders and shows a pronounced minimum at harmonic 17, as was previously observed at a lower laser intensity^27^. Varying the laser intensity in our experiment does not modify the position of this minimum.

The intensity of the harmonic emission at frequency *ω* for channel *j* is determined by the SFI probability 
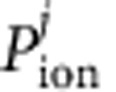
, the energy spectrum of the recolliding electron wavepacket in the continuum *S*_elec_^*j*^(*ω*) and the recombination cross section 
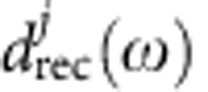
11,12: 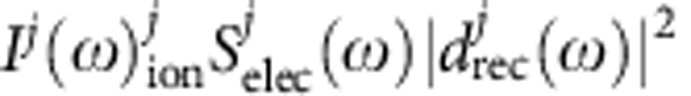. We assume that the recolliding electron wavepacket spectrum has the same energy structure for all channels as in argon since they have similar ionization potentials: 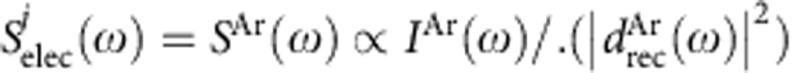. Using the measured harmonic spectrum and the experimental photoionization cross section of Ar from ref. 28, we obtain the wavepacket spectrum. Normalizing the harmonic spectrum of SF_6_ by *S*_elec_(*ω*) provides a quantity which is only related to ionization probabilities and recombination cross-sections: 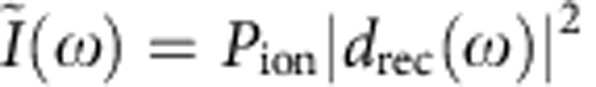. This normalized spectrum shows a maximum at harmonic 15 and a minimum at harmonic 17 (Fig. 3b).

Calculating the high-order harmonic spectrum from SF_6_ is quite challenging due to the number of associated channels and the presence of resonances in some of them. As a first approximation, we assume that there are no subcycle non-adiabatic multi-electron transitions, which would coherently couple cation states: an electron leaving a given cation electronic state will recombine to the same state. We will not try to simulate the harmonic emission resulting from the coherent superposition of different channels, but simply to estimate the contribution of each channel.

We make an approximation that the recombination dipole moment 
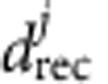
 is equal to the field-free XUV photoionization dipole moment. This was studied experimentally using synchrotron radiation, but the A and B channels are not distinguished^29^. We thus prefer to rely on theoretical calculations, which provide differential cross-sections for all channels. Modelling the photoionization cross-sections of SF_6_ is difficult and requires taking into account accurately intra- and interchannel couplings^30^,^31^ (Supplementary Fig. 2). The recent calculations from Jose *et al*. show good agreement with experiments, once downshifted by 2 eV (Supplementary Fig. 3). The normalized harmonic intensity 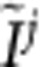 calculated for each channel using these calculated cross-sections 
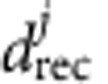
 and the calculated 
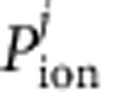
 from Fig. 2b is shown in Fig. 3b. The picture, which is very complex in the case of XUV photoionization where many channels have similar cross-sections (Supplementary Fig. 3), appears to be simplified by SFI. The tunnelling probability from the ground-state channel X is significantly reduced by the presence of nodes in the corresponding orbital, such that the deeper A channel is favoured.

Below 26 eV, there are broad maxima in the cross-sections from A, B and C channels, which significantly increases their contributions. These maxima are related to the following shape resonances as calculated by Jose *et al*.: 5*t*_1*u*_→*εt*_2*g*_ (A channel-40 Mbarn) at 24 eV, 1*t*_2*u*_→*εt*_2*g*_ (B channel-90 Mbarn) as well at 24 eV and finally the 3*e*_*g*_→*εt*_1*u*_ (C channel-15 Mbarn) at 22 eV (ref. 31, see Supplementary Discussion for details). Due to the coupling between X and (A, B) channels, a slight enhancement in the cross-section at the energy of the shape resonance is as well observed in the X channel (37 Mbarn)^31^. Last, another broad peak is predicted in the A channel around 38 eV, associated to the 5*t*_1*u*_→*εe*_*g*_ shape resonance (40 Mbarn).

Comparison with the measured harmonic signal (crosses in Fig. 3) indicates that the maximum at H15 may be the signature of the 5*t*_1*u*_→*εt*_2*g*_ shape resonance in the A channel. By contrast, there is no clear signature around H25 of the resonance predicted at 38 eV (5*t*_1*u*_→*εe*_*g*_) in the harmonic spectrum. Noteworthy, experimental photoionization cross-sections hardly show any feature related to this resonance either^29^. However, the presence or absence of a maximum in the harmonic spectrum is insufficient to draw any definitive conclusion on the involvement of resonances in the process^16^.

### Revealing resonant HHG using elliptical laser light

While the qualitative study of harmonic spectra has indicated the possible role of the 5*t*_1*u*_→*εt*_2*g*_ shape resonance in the emission, a definitive assignment of the structures observed in the experimental spectrum would require a full calculation of the emission, taking into account the complex photorecombination cross-sections and including the effects of laser-induced couplings. How can we identify the presence of resonances without needing a full theoretical model of the harmonic emission? Such identification requires a differential measurement, a measurement that reveals the response of the molecular system to a variation of the laser parameters. This can be achieved by measuring the variation of the harmonic signal with laser ellipticity, *ε*.

High-order harmonic emission in an elliptical laser field can be described in a quasi-static semi-classical picture, as follows. First, electrons tunnel out of the molecule with a finite transverse momentum distribution. This distribution is dictated by the molecular orbital structure, filtered through the tunnelling process^32^. If the electron wavepacket at the exit of the tunnel is spatially localized, then the momentum distribution is broad. During propagation in the continuum, the electron wavepacket spreads transversely. The spread of the returning electron wavepacket depends on the time spent in the continuum (and thus on the harmonic order, the lowest harmonics being emitted before the highest ones^33^), but also on the initial transverse momentum distribution: the broader the initial transverse momentum distribution, the broader the returning wavepacket. With an elliptically polarized driving field, the orthogonal component of the field shifts the electron in the lateral direction, leading to a suppression of the recollision probability. Such suppression depends on both the trajectory length and the initial channel-specific transverse momentum.

**Figure 4:**
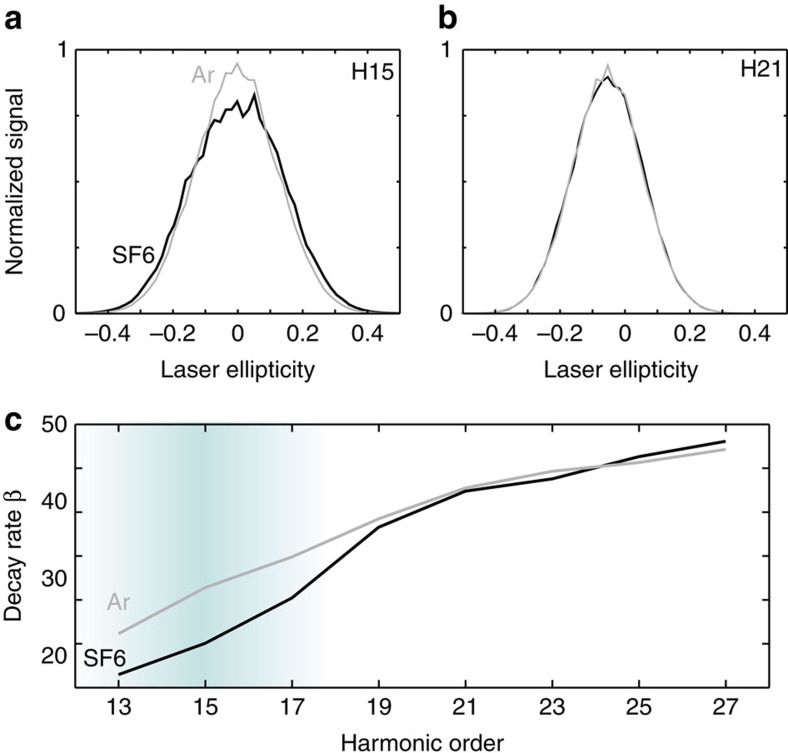
HHG in elliptical laser fields. Intensity of harmonic 15 (**a**) and 21 (**b**) generated in Ar (grey) and SF_6_ (black) at *I*≈1.3 × 10^14^ W cm^−2^ as a function of ellipticity. (**c**) Decay rate β of the harmonic signal with ellipticity as a function of harmonic order.

The measured harmonic signal as a function of laser ellipticity in argon and in SF_6_ is shown in Fig. 4a,b. The harmonic intensity decays exponentially with *ε*^2^ (ref. 34) and can be fitted by a Gaussian 
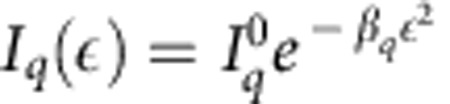
, where *q* is the harmonic order and *β*_*q*_ the variation with ellipticity. The ellipticity-dependent responses of argon and SF_6_ are remarkably similar for harmonic 21, whereas that of argon is higher for harmonic 15. The variation of the decay rate β with harmonic order is shown in Fig. 4 for Ar and SF_6_(c). A clear transition is visible about harmonic 17: the harmonic signal decays slower in SF_6_ in the lower energy range.

The trajectory lengths are similar in the Ar and SF_6_ measurements (same intensity, close ionization potentials). The similarity of the decay rates with ellipticity in these two species above harmonic 17 shows that the transverse momentum distributions of the involved channels are very similar. Calculations of the momentum distributions indeed give identical results for Ar and the A channel (Supplementary Fig. 4). This measurement thus tends to indicate the predominance of the A channel in the emission above H17. The sudden transition to a regime of lower decay rate with ellipticity below H17 shows a switch in the generating process. This could either be due to a transition to another ionizing state, or more likely to the influence of the resonance in recombination.

**Figure 5:**
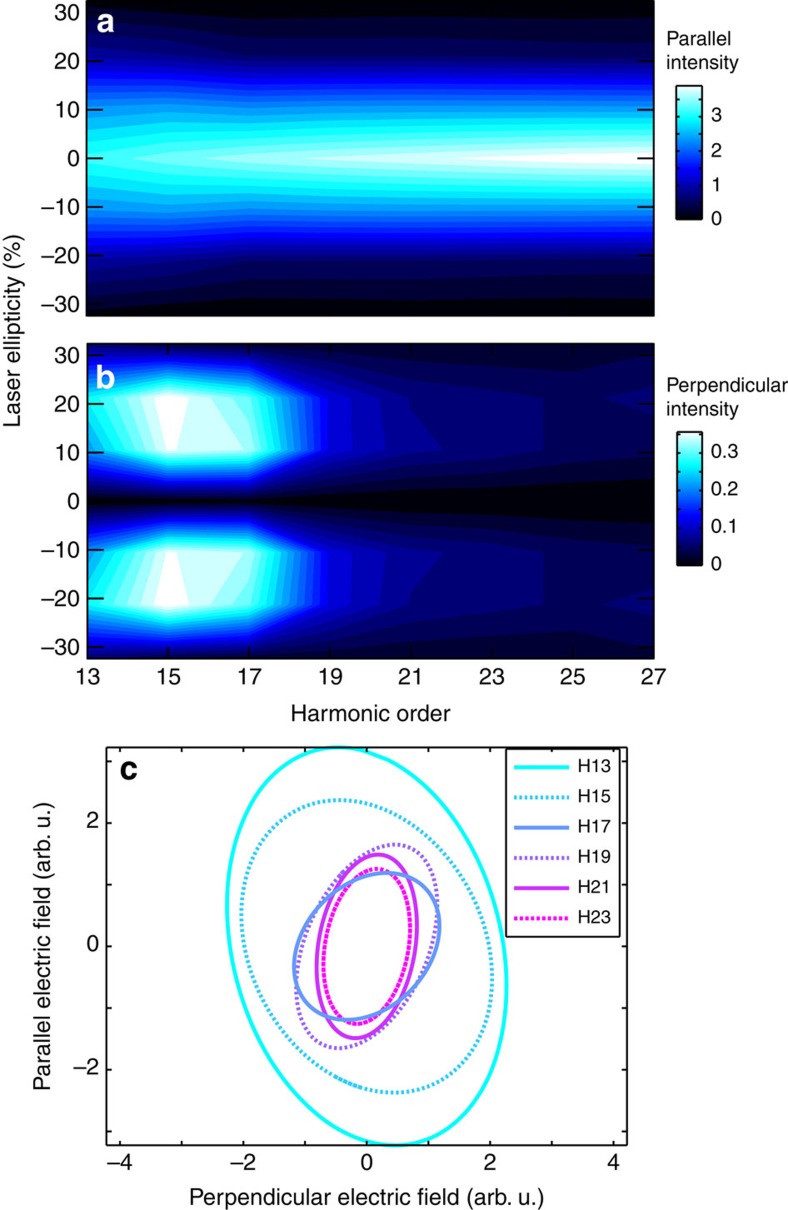
High-harmonic polarimetry. Parallel (**a**) and perpendicular (**b**) components of the harmonic emission as a function of laser ellipticity. (**c**) Polarization ellipses of the harmonics generated by a 20% elliptical laser field.

The high-order harmonic emission from an elliptical laser field is a vectorial quantity: the harmonic electric field can be decomposed in two projections, parallel and perpendicular to the main axis of the fundamental ellipse. Experimentally, these two projections are resolved by introducing an XUV polarizer between generation and detection. Rotating the polarizer (or equivalently the fundamental ellipse) enables measuring modulations of the harmonic signal, which are characteristic of the Malus law^35^. Assuming that the radiation contains no unpolarized component, we can extract the polarization state of the harmonics as a function of the laser ellipticity, and therefore the evolution of the two projections of the harmonic signal. The results are shown in Fig. 5a,b. The signal of each harmonic is normalized by the sum of the parallel and perpendicular intensities over all ellipticities. The lower decay rate of the harmonic signal with ellipticity is accompanied by the creation of a strong orthogonal component. Figure 5c shows the polarization ellipses of the harmonics obtained with a fundamental ellipticity *ε*_0_=20%. Harmonic 13 to 17 show high degrees of ellipticity and the direction of the polarization ellipse changes sign between H15 and H17. These features were found to be robust against variation of the laser intensity.

We have recently performed a theoretical and experimental study of HHG in Ar atoms, which revealed that resonances could induce strong orthogonal components of the harmonic emission and high degrees of ellipticity^36^. This study also confirmed that the radiation emitted by SF_6_ in the 20–25 eV range was not unpolarized but highly elliptical. The polarization-resolved measurements presented in Fig. 5 thus constitute a clear demonstration of the contribution of a resonance in the HHG process for harmonic 13 to 15. Comparison with the calculations of Fig. 3b enables us to assign this resonance as the 5*t*_1*u*_→*εt*_2*g*_ shape resonance in the A channel. We also note that there is no sign of the other shape resonance (5*t*_1*u*_→*εe*_*g*_) predicted around H25 in Fig. 3b.

### Measurement of the recombination phase

Elliptically driven HHG in SF_6_ revealed a clear transition in the emission process: harmonics 13 to 17 are produced by resonant emission, probably mostly through the A channel, whereas harmonics above 17 are produced by non-resonant emission. This resonance is expected to induce a phase shift in the emission but to the best of our knowledge no data is available on this matter in the litterature. Indeed, measuring phase shifts in photoionization matrix elements is a complicated task. Attosecond pulse trains can be used to determine such shifts, the main difficulty being the decyphering of photoelectron spectra containing many components^37^. By contrast, measuring the phase shift in the harmonic emission is quite straightforward.

**Figure 6:**
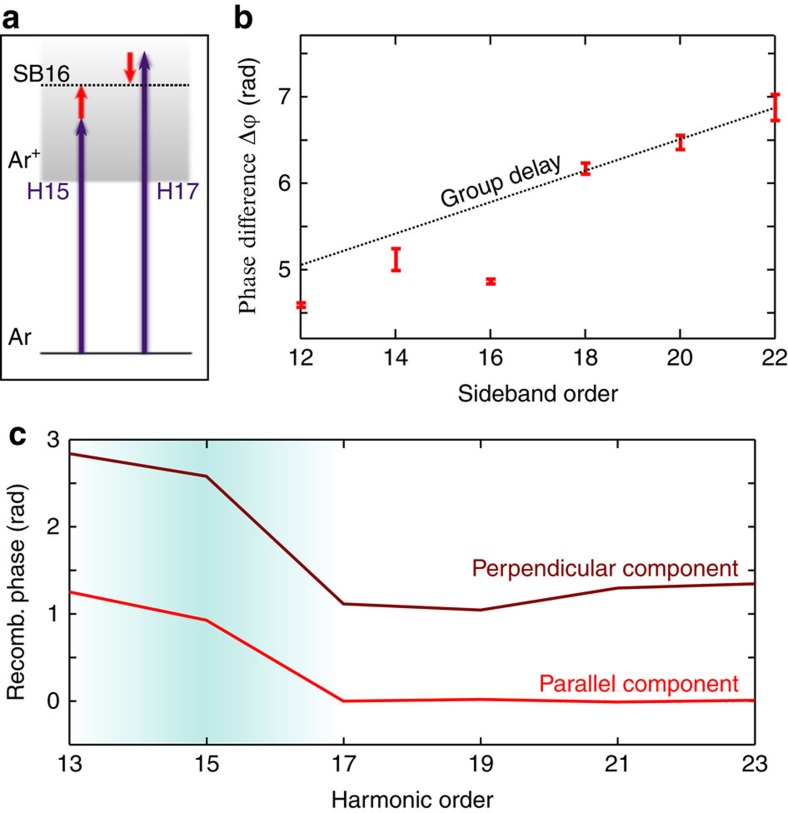
Measurement of the harmonic phase. (**a**) Principle of the RABBIT method: high harmonics photoionize argon atoms in the presence of an infrared (800 nm) field. Absorption of harmonic 15 and one IR photon or absorption of harmonic 17 and stimulated emission of one IR photon both lead to the production of the same sideband (SB16) in the photoelectron spectrum. (**b**) Phase of the sideband oscillations as a function of the delay between IR and harmonics. The dotted line is the linear contribution of the group delay dispersion. (**c**) Recombination phase as a function of harmonic order (light), and recombination phase of the perpendicular (dark) component of the harmonic emission in a 20% elliptical laser field.

The RABBIT technique^38^ is an attosecond pulse characterization method that measures the relative phase between consecutive harmonics. RABBIT is based on photoionization of a target gas by high-order harmonics, in the presence of a weak fundamental laser field. The photoelectron spectrum shows main lines, resulting from the absorption of harmonics, and sidebands, corresponding to absorption of one harmonic and absorption or emission of an infrared photon (Fig. 6a). Two quantum paths involving two consecutive harmonics lead to the same sideband, which results in an interference process. The sideband intensity thus oscillates as a function of delay between harmonics and the IR. The phase of this oscillation 
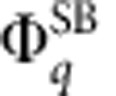
 encodes the relative phase between the neighbouring harmonics *q*+1 and *q*−1: 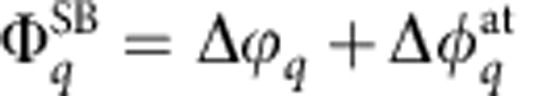, where 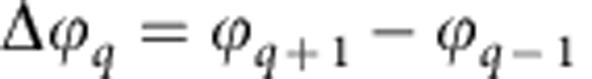 is the phase difference between harmonics *q*+1 and *q*−1 and 
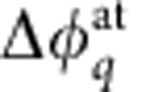
 is a scattering phase intrinsic to the target (the atomic phase, that is, the phase accumulated by the photoionized electron in the atomic scattering potential^38^).

In Fig. 6b, we show the measured harmonic phase difference obtained using SF_6_ as a generating gas and argon as a detecting gas. In a simple single-channel non-resonant emission case, the harmonic phase difference is expected to increase linearly with harmonic order, reflecting the group delay dispersion of the attosecond pulse emission. This linear behaviour is visible on the three highest sidebands. A clear deviation is observed at sideband 16, reflecting a specific phase difference between harmonic 15 and 17. A similar behaviour was reported in ref. 39. We decompose the harmonic phase as the sum of a continuum phase, reflecting the group delay and a recombination phase: 
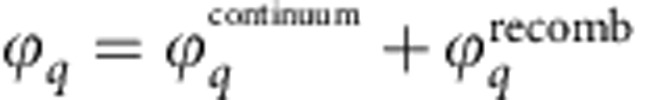
. Integrating the phase difference after subtracting the linear contribution of the continuum phase, we obtain the recombination phase plotted in Fig. 6c (for the parallel component). There is a 1.2-rad phase jump through the resonance, with the major part (1 rad) between H15 and H17.

The RABBIT measurement was performed in linear laser polarization and thus measured a phase jump in the parallel recombination dipole moment. This phase jump occurs at the same position as the sign change in the polarization ellipse direction (Fig. 5c), which indicates a phase jump also in the orthogonal dipole moment. Assuming that the phase of the parallel dipole moment does not vary as the laser ellipticity increases from 0 to 20%, one can combine the polarimetry and RABBIT measurements to extract the recombination phase of the perpendicular component (by adding the relative phase between the two vectorial components obtained in polarimetry to the phase of the parallel component obtained in RABBIT). The results, shown in Fig. 6c, show a larger phase jump (1.7 rad) across the resonance in the perpendicular component. This determination of the recombination phase along the two components of the harmonic emission demonstrates the strength of the combination of RABBIT and polarimetry measurements.

### Response to molecular vibrations

We have identified a transition between two regimes in HHG from SF_6_—resonant (below H17) and non-resonant (above H17)—by manipulating the electron trajectories in the generating mechanism. The emission through a shape resonance involves a peculiar process of electron scattering within the molecular potential, which is expected to be sensitive to changes of the molecular geometry. In the following, we vary the nuclear degrees of freedom via vibrational excitation. As discussed below, this further highlights the specific nature of the resonant HHG process.

Small-molecular structural changes in the generating medium can induce significant modifications in the high-harmonic emission. For instance, Raman pump—strong-field probe experiments in SF_6_ showed that the intensity of harmonic 25 to 45 varies by ~10% on molecular vibration^40^,^41^, even if the bond length variations remain small (~1%). First, changes in molecular geometry are expected to alter the vertical ionization potentials. Such modification influences the relative amplitudes and phases of the channels. For example, a 65-meV change in *I*_p_ results in a 100-mrad phase change for a plateau harmonic. Second, molecular vibrations can lift the electronic degeneracy of intermediate cation states, increasing the number of distinct channels. Finally, any distortions of the electronic wavefunctions associated with these vibrations may affect both the ionization and recombination dipole moments, in particular in the vicinity of resonances.

**Figure 7:**
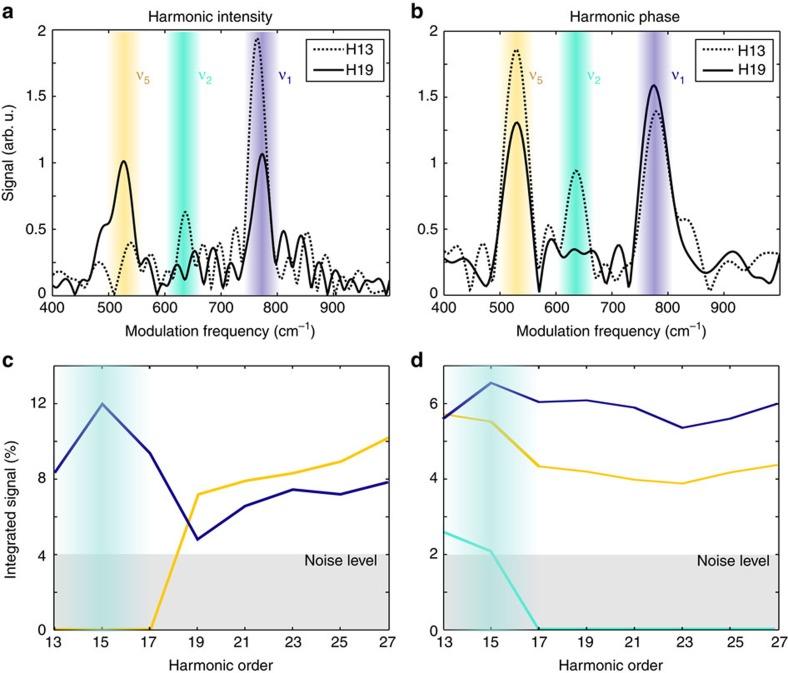
The modulation of HHG by specific vibrations in ground-state SF_6_ molecules. Fourier transform of the intensity (**a**) and phase (**b**) modulations with pump-probe delay for harmonic 13 (dots) and 19 (line) showing the contribution of different Raman modes: *ε*_5_−T_2*g*_ (524 cm^−1^, yellow), *ε*_2_−E_*g*_ (643 cm^−1^, green) and *ε*_1_−A_1*g*_ (774 cm^−1^, blue). The contribution of each mode, obtained by integration of the peak in the FFT, is shown as a function of harmonic order in (**c**) intensity and (**d**) phase.

We have performed pump-probe experiments using an intense (~7 × 10^13^ W cm^−2^) 800 nm pulse to Raman-pump vibrational modes in the SF_6_ molecules and produce high harmonics in the excited medium. In addition to conventional experiments in which the harmonic intensity is measured (Fig. 7a,c), we have used two-source interferometry to determine the evolution of the harmonic phase with pump-probe delay (Fig. 7b,d)^5^,^42^ (see Methods). The harmonic emission is periodically modulated as a function of pump-probe delay. The typical modulation depth of the harmonic signals are 5% for the intensity and 100 mrad for the harmonic phase. Three of the six normal modes of SF_6_ are Raman active vibrations: *ε*_1_-fully symmetric and strongly active mode *A*_1*g*_ with a quantum of 774 cm^−1^ (vibrational period of ≈43 fs), *ε*_2_-the doubly degenerate mode *E*_*g*_ with a quantum of 643 cm^−1^ (≈52 fs) and finally the triply degenerate *ε*_5_−*T*_2*g*_ mode with a quantum of 524 cm^−1^ (≈63 fs)^43^. A Fourier analysis is performed to determine which vibrational modes modulate the signal (Fig. 7a,b).

The weight of each vibrational mode in the intensity and phase modulations is extracted by integrating the corresponding peak and normalizing to the total signal (Fig. 7c,d). The results show a clear transition around harmonic 17, consistent with the switching from resonant to non-resonant emission measured in static molecules. Below harmonic 17, in the region of the shape resonance, the harmonic intensity is exclusively modulated by the *ε*_1_ mode. Clearly the asymetric distorting mode *ν*_5_ does not impact the amplitude of the harmonic emission through the shape resonance. Above harmonic 17, the *ε*_5_ mode becomes slightly predominant. There is no significant contribution of the *ε*_2_ mode except for harmonic 13. Interestingly, the behaviour of the harmonic phase is very different but also shows a transition. The phase of the resonant emission varies similarly for *ε*_1_ and *ε*_5_ nuclear dynamics and shows a weak contribution from *ε*_2_. The phase of the emission above H17 seems insensitive to *ε*_2_ and dominated by *ε*_1_. These results represent to our knowledge the first dynamical study of a shape resonance in HHG. Shape resonances are known to depend on bond length^44^ and our results reflect this dependence as the molecule vibrates.

## Discussion

Multi-channel HHG in polyatomic molecules is a rich and complex process that initiates a broad range of multi-electron phenomena. The identification and isolation of the different channels is a major challenge, which requires the development and integration of new experimental tools. In this work we introduced and combined several experimental schemes in an advanced spectroscopic study, enabling us to elucidate clear features related to resonant and non-resonant HHG in SF_6_. First, we identified the appearence of different SFI channels by performing wavelength-dependent fragment-resolved ATI measurements. Varying the wavelength addressed the issue of overlapping ATI combs that can occur at specific wavelengths, notably at the 800-nm driving frequency implemented in the majority of this work. We introduced a new scheme to HHG spectroscopy by resolving the spectral dependence of the normalized ellipticity response. In non-resonant cases, this measure enables isolation of the transverse shape of the electronic wavefunction at the moment of tunnel ionization, removing the complexity imposed by the additional steps of the interaction. In resonant cases, the laser ellipticity induces the creation of a strong orthogonal dipole moment and a large ellipticity of the harmonic emission, which were detected by polarimetry. We made use of the coherent properties of the process via RABBIT measurements, which enabled the measurement of the recombination phase through the resonance. Finally, we have made use of the nuclear degrees of freedom—via vibrational dynamics—as an important control knob that manipulates both the phase and amplitude of the emission, advancing our ability to identify the specific nature of the resonant generation process.

All the experimental observations lead to the assignment of the 5*t*_1*u*_→*εt*_2*g*_ shape resonance in the A channel as the main contributor to the generation of harmonics 13 to 17. This result is particularly striking compared with photoionization experiments in which it is the resonance in the B channel that dominates the process. In HHG, this channel is suppressed by the inefficiency of strong-field ionization due to a large number of nodal planes in the orbital profile. No feature related to the 5*t*_1*u*_→*εe*_*g*_ shape resonance in the A channel was observed in any of the measurements. This absence, which is consistent with the very weak contribution of this resonance to experimental photoionization cross-sections, calls for additional theoretical studies. In particular, Jose *et al*.^31^ suggested that vibrational excitation could drastically affect the shape resonance. We believe that high-harmonic spectroscopy is the ideal tool to study such effects. On one hand, the vibration of the neutral can be controlled using stimulated impulsive Raman scattering as we have shown. On the other hand, the dynamics of the ion between ionization and recombination are encoded in the harmonic spectrum^45^, and can be probed by measuring the wavelength dependence of the harmonic signal. Last but no least, the coherence of the HHG process enables performing phase-resolved measurements and accessing the temporal properties of the emission, observing the decay of autoionizing states in the time domain.

The identification of the basic mechanism associated with multiple channels is the first fundamental step in characterizing time-resolved multi-electron processes, the long term goal being to resolve charge migrations or field-induced coupling between the channels. We believe that it will be possible to isolate such dynamics by increasing the dimensionality of the experiment by scanning the fundamental wavelength of the strong laser field in HHG. Such scanning will scale the interferometer, presented in Fig. 1, enabling the decoupling of the different degrees of freedom involved in the process. In summary, we believe that the combination of methods presented here constitute an important building block for strong-field spectroscopy and will lead to the discoveries of a broad range of multi-electron strong-field phenomena in complex targets such as polyatomic molecules.

## Methods

### Fragment-resolved ATI

Experiments were performed using a Legend Elite Duo laser system (Coherent Inc.) that delivered 35 fs, 3.1 mJ, 800 nm pulses at 1 kHz (Molecular Photonics Laboratory, NRC Ottawa). Laser fundamental pulses were implemented directly in the 800-nm experiments, whereas ~40-fs 400-nm pulses were generated through second harmonic generation in thin β-BaBO_4_ crystals (150-μm thick). Approximately, 40 fs 480, 532 and 590 nm pulses were generated through sum frequency generation of the signal pulses from an optical parametric amplifier (Light Conversion, TOPAS-C) and the fundamental beam in thin β-BaBO_4_ crystals (250 μm thick). Laser pulses were focused with a f=50 cm spherical mirror onto a continuous, neat expansion of SF_6_ molecules (expanded through a 50-μm pinhole ~60 cm from the laser–molecular beam interaction region in a differentially pumped chamber). The molecular beam PEPICO (PhotoElectron PhotoIon COincidence) spectrometer used to collect the resulting photoelectrons and photoions has been described elsewhere^23^. In brief, the kinetic energies of the photoelectrons were resolved by time of flight using a wide-bore permanent magnet, magnetic bottle spectrometer. Photoions were collected using a coaxial pulsed-field Wiley–McLaren time-of-flight mass spectrometer. The photoelectron and photoion time-of-flight signals were post processed using a covariance analysis^46^,^47^ to correlate specific electron signals with the dominant 
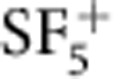
 ions. The typical exponential ATI decay in energy has been subtracted for clarity. The energy and intensity calibration is a multi-step process, which uses the ATI progressions themselves as a ruler, and also involves investigating the intensity-dependent ATI spectra of O_2_ and H_2_O impurities, where Stark shifts can be neglected.

### Calculations of SFI rates

Bound-state neutral and cation wavefunctions were calculated using aug-cc-pVTZ basis set^48^, large-core effective core potentials^49^ and RASSCF wavefunctions with 35 (cation) or 36 (neutral) electrons in 25 orbitals, allowing single and double excitations. For the cation, the active space was optimized for an equally weighted average of the six lowest states of the cation (X through D). The RASSCF vertical IPs were shifted uniformly by −0.512 eV. At equilibrium, *O*_*h*_ geometry (R(S-F)=1.575 Å), the resulting cation state energies are within 0.3 eV of the correlated MCQDPT2(27/28,21)/aug-cc-pVTZ values.

The magnitude of the vibrational distortions induced by an impulsive Raman pump was determined^50^ from normal vibrational modes and dipole polarizabilities calculated at the CCSD/aug-cc-pVTZ level^51^. Calculated displacements assumed pump energy flux of ≈8.4 J cm^−2^. Pump and probe polarizations were assumed to be collinear and were integrated on an order-9 Lebedev grid^52^. RASSCF and MCQDPT2 (ref. 53 calculations were performed with GAMESS-US^54^. CCSD calculations used CFOUR^55^. Strong-field ionization calculations^25^,^26^ used Cartesian product grid with 0.18 Bohr spacing, extending to ±18 Bohr from the origin, with an absorbing boundary^56^ of 9.4 Bohr at the box edges. The simulation used 14 spatial channels, corresponding to the X, A, B, C and D final states of the cation. Both laser and correlation coupling terms were included. Leap-frog time evolution used a time step of 0.003 AU.

### High-order harmonic spectroscopy

The high-harmonic spectroscopy experiments were performed using the Aurore laser system at CELIA, which delivers 28 fs, 7 mJ, 800 nm pulses at 1 kHz. High harmonics were generated by focusing the pulses with a *f*=50 cm lens in a continuous jet produced by a 250-μm nozzle backed by a few hundred mbars of gas. The emitted radiation was analysed by an extreme-ultraviolet spectrometer (1,200 mm^−1^ grating, dual Micro Channel Plates and a CCD camera).

To manipulate the laser ellipticity, two broadband zero-order waveplates were inserted in the laser beam: a motorized half waveplate in front of a fixed quarter waveplate. The harmonic signal was recorded as a function of the angle *α* between the half and quarter waveplate axis that determines the laser ellipticity *ε*=tan (2*α*). The harmonic polarization state was characterized using an XUV polarizer consisting of three bare gold mirrors under 75–60–75° incidence, which leads to a typical contrast of 10–20 between S and P polarizations in the studied spectral range. A second half-wave plate was used to rotate the laser polarization, which is equivalent to rotating the XUV polarizer, to record the Malus’ law.

For RABBIT measurements, we used the experimental set-up described in ref. 57. In brief, the harmonic and infrared beams were delayed using a beam-splitting dichroic delay-line. They were focused by a toroidal mirror in a magnetic bottle time-of-flight spectrometer that recorded the electron spectrum produced by photoionizing argon atoms, as a function of delay between XUV and infrared beams.

Phase-resolved pump-probe experiments were performed using a 0–π phase mask^42^ inserted on the harmonic generating beam, before focusing^5^,^58^,^59^. This mask created an interference pattern at focus, which resulted in the creation of two harmonic sources spatially separated by ~100 μm. In the far field, these sources produced an interference patern, similar to Young’s slits. An 800-nm pump beam was superimposed on one of the two sources, exciting the molecules by impulsive stimulated Raman scattering. The evolution of the phase of the harmonic emission as a function of pump-probe delay was measured by determining the shift of the interference fringes, using a spatial Fourier transform.

## Author contributions

A.E.B., J.M. and I.W. carried out the strong-field ionization measurements, S. Patchkovskii the strong-field ionization calculations, V.B., A.F. and Y.M. the high-harmonic polarimetry measurements, N.F., J.G., G.G. andf Y.M. the RABBIT measurements, V.B., B.D.B, A.C., M.D., A.F., Y.M., T.R., H.S. and D.S the molecular vibration measurements. F.B., D.D. and S. Petit operated the laser system at CELIA. B.F., A.F. and Y.M. analysed the high-harmonic data. A.E.B., J.M., S. Patchkovskii, A.S. and I.W. analysed the strong-field ionization data. N.D. and Y.M. designed the manuscript. All authors contributed to the interpretation of the data and writing of the manuscript.

## Additional information

**How to cite this article:** Ferré, A. *et al*. Multi-channel electronic and vibrational dynamics in polyatomic resonant high-order harmonic generation. *Nat. Commun.* 6:5952 doi: 10.1038/ncomms6952 (2015).

## Supplementary Material

Supplementary Information Supplementary Figures 1-4, Supplementary Discussion and Supplementary References
