# Supplementary material for: Multi-channel electronic and vibrational dynamics in polyatomic resonant high-order harmonic generation
Source: Nat Commun. 2015 Jan 22;6:5952. doi: 10.1038/ncomms6952 (PMC4354235; doi:10.1038/ncomms6952)
Supplement: Supplementary Information — Supplementary Figures 1-4, Supplementary Discussion and Supplementary References [file ncomms6952-s1.pdf]

I. SUPPLEMENTARY FIGURES

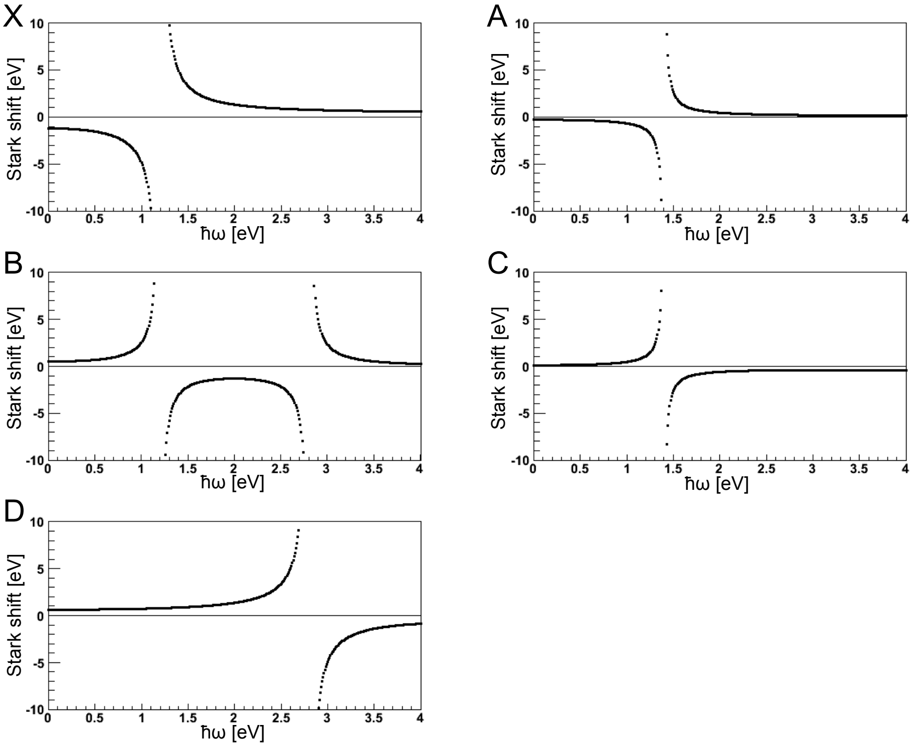

Supplementary Figure 1: Stark shifts for the different SFI channels in SF_6 at an intensity of 5e13 W/cm^2, calculated using the method described in the text.

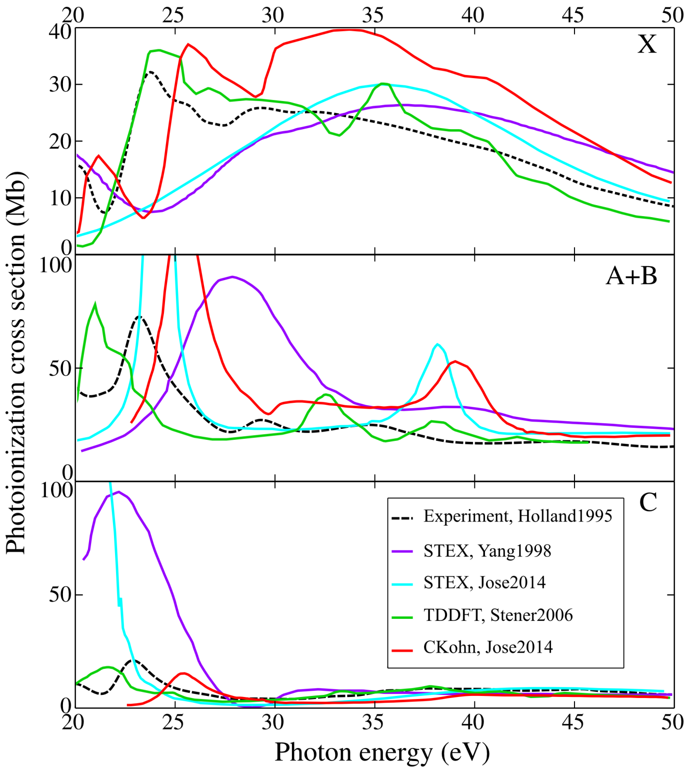

Supplementary Figure 2: Photoionization cross sections of SF_6 for channels X (top), A+B (middle) and C (bottom). Experimental data from [3] (black dashed line) and results from different calculations are shown: STEX from [4] (purple), STEX from [5] (blue), TDDFT from [6] (green) and CKohn from [5] (red).

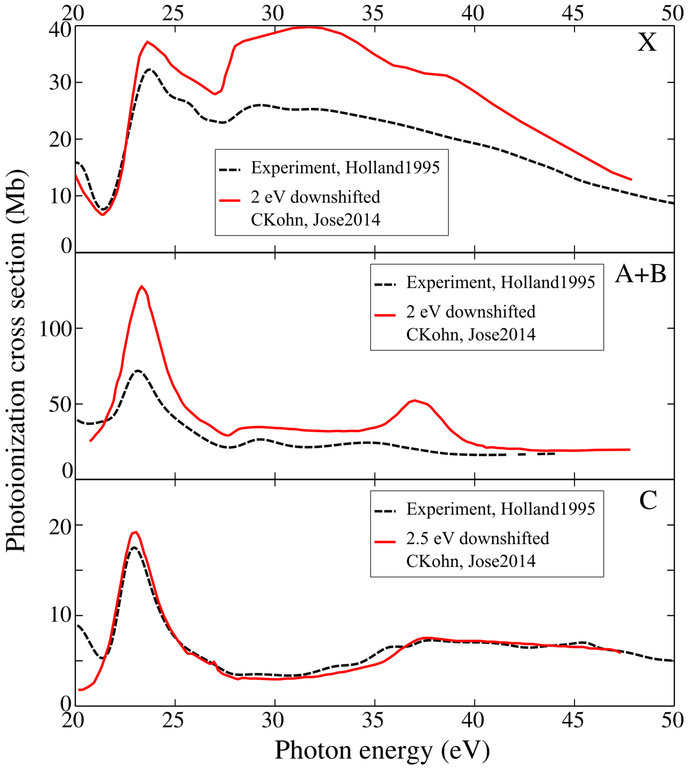

Supplementary Figure 3: Comparison between experimental (black dashed) [3] and energy-shifted theoretical cross sections calculated by Jose *et al.*(red) [5]. For the X, A and B channels the cross theoretical cross sections have been horizontally transleted by 2 eV. For the C channel the translation is 2.5 eV.

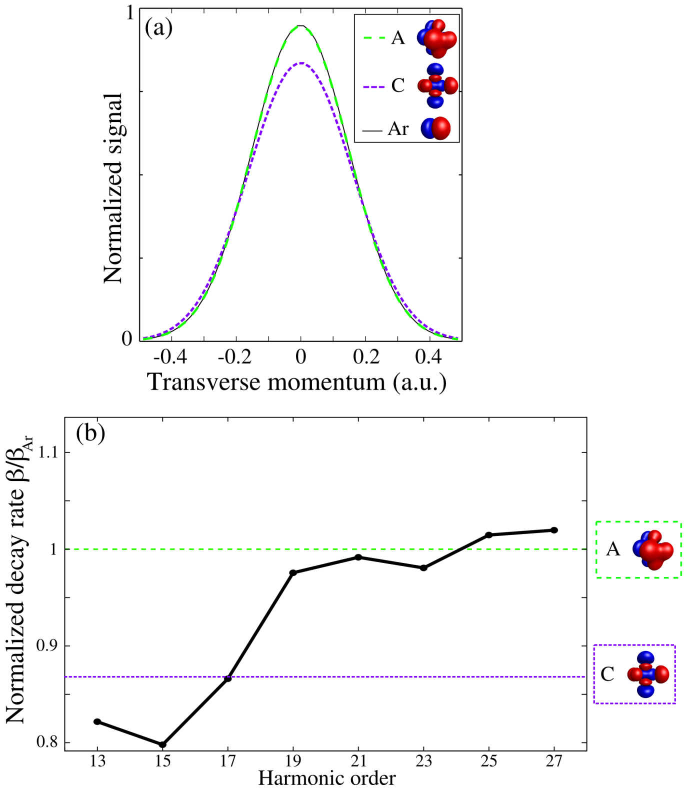

Supplementary Figure 4: (a) Calculated electron transverse momentum distribution in argon and the A and C channel from SF_6. (b) Measured response of the harmonic signal as a function of laser ellipticity in SF_6, normalized to that in Ar. The horizontal dashed lines are the theoretical normalized ellipticity-dependent responses for the A (green) and C (purple) channels, respectively.

II. SUPPLEMENTARY DISCUSSION

A. State-specific frequency-dependent Stark shifts in SF_6

Since the SF_6 molecule does not have a permanent dipole moment, the lowest order Stark effect is quadratic in the field, written in cycle-averaged form as

$$\Delta E_{\mathrm{Stark}} = -\frac{1}{4}\alpha F_0^2, \tag{1}$$

where F_0 denotes the peak electric field and α the polarizability, averaged over all orientations of the unaligned molecule. Higher order effects have been neglected. Since what matters in the experiment are the differential Stark shifts between the neutral state and the different ionic states, we have to determine state-specific polarizabilities. Moreover, since in the SF_6^+ cation excited states are quite closely spaced, the frequency dependence of α is expected to be significant.

Polarizability calculations were performend at the optimized MP2/aug-cc-pVTZ geometry (R(S-F)=1.5750 Angstrom). Frequency-dependent polarizabilities of the neutral species were calculated directly, using EOM-CCSD/aug-cc-pVTZ method as implemented in CFOUR v2 [1]. As expected for a neutral, closed-shell molecule, SF_6 polarizability frequency dependence is negligible for wavelengths below 400 nm.

In contrast, the SF_6^{1+} cation possesses several electronic states within the 5 eV of the electronic ground state, leading to a strong frequency dependence of polarizability in the visible to near-IR wavelength range. Unfortunately, it has proven impossible to evaluate frequency-dependent polarizability of the spatially-degenerate cationic states using EOM-CCSD and EOM-IP-CCSD approaches available in the current version of CFOUR v2. Instead, we *estimate* the frequency-dependent polarizabilities as outlined below.

Formally, frequency-dependent polarizability of an electronic state i is given by a sum-over-states expression:

$$\alpha_i(\omega) = 2\sum_{j\neq i}^{\infty} |\langle\Psi_i|r|\Psi_j\rangle|^2 \frac{(E_j - E_i)}{(E_j - E_i)^2 - (\hbar\omega)^2}, \quad (2)$$

where summation is in principle over all electronic states, including resonances and continuum states. In practice, direct applications of Eq. 2 require summation over thousands of states, which is out of question for a molecule of SF_6's electronic complexity.

Instead, we choose to treat the first few terms in Eq. 2 explicitly, and estimate the remaining terms from the calculated finite-field static polarizabilities $\alpha_i(0)$. Specifically:

$$\alpha_i(\omega) \approx \alpha_i(0) + 2\sum_{j\neq i}^{N_{\max}} |\langle\Psi_i|r|\Psi_j\rangle|^2 \left[\frac{(E_j - E_i)}{(E_j - E_i)^2 - (\hbar\omega)^2} - \frac{1}{E_j - E_i}\right] \quad (3)$$

In eq. 3, static polarizabilities of the cationic states were calculated using finite-field restricted active-space SCF (RASSCF) wavefunctions and aug-cc-pVTZ basis set. The active space contained 18 occupied (a_{1g}, e_g, t_{1g}, t_{2g}, 2× t_{1u}, and t_{2u} irreducible representations) and 7 virtual (a_{1g}, t_{1u}, and t_{2g} irreps) molecular orbitals, with single and double excitations allowed between the two spaces. The RASSCF calculation was averaged over 14 equally-weighed electronic states, encompassing spatial components of the X, A, B, C, and D states of the SF_6^{1+} cation. The static electric field was $F = 0.001$ atomic units. Electronic states and transition dipoles between the lowest 37 states of the cation ($N_{\max} = 37$) were calculated using MR-CIS wavefunctions, allowing single excitations from the RASSCF reference above to further 24 molecular orbitals. GAMESS-US [2] code was used for electronic structure calculations.

B. Photoionization cross sections of SF_6

The matrix elements governing the radiative recombination of an electron and its parent ion in HHG are very similar to the ones governing photoionization of a neutral molecule. In this part we review the results of a few calculations of the photoionization cross sections of SF_6 to explain the choice that has been made for the calculations of HHG signals.

The photoionization cross sections of SF_6 have been measured by Holland *et al.* in 2005 using synchrotron radiation [3]. Channels A and B were not resolved in the experiment due to their proximity in energy. The results show several resonant features, in particular a peak around 23 eV in the A+B and C cross sections (Suuplementary Figure Supplementary Figure 2). The results of different theoretical studies are shown for comparison in Supplementary Figure Supplementary Figure 2. Two of these studies are based on the statix exchange approximation (STEX), including full intrachannel interaction but no interchannel interaction [4, 5]. They both produce very large resonant peaks in the A+B and C channels. The position of these peaks differs depending on the applied model. Furthermore, the resonances in channels A+B are located at higher energies than in channel C.

The last two calculations do include interchannel couplings, either by using the Time-Dependent Density Functional Theory (TDDFT, [6]) or complex Kohn variational technique (cKohn, [5]). The overall agreement with experimental cross sections is much better. The calculated cross sections still show peaks associated to resonances in the A,B and C channels, but they are weaker and all located at the same energy: ∼ 22 eV for TDDFT and ∼ 25 eV for CKohn.

In order to estimate the contribution of each channel to HHG, we need to distinguish the A and B channels, whose strong-field ionization yields are very different. We thus chose to rely on theoretical cross sections rather than on experiments. We used the CKohn results from [5] because in the TTDFT results only the total cross section of A+B has been published.

Even though the CKohn results are in decent overall agreement with the experiment, there is a clear slight shift in energy between the two. In Supplementary Figure Supplementary Figure 3 we corrected this shift by translating the theoretical results down by 2 eV for the X, A and B channels and by 2.5 eV for the C channel. The agreement with experiment is very good. Two main discrepancies can be noted. First, the resonant peak at 23 eV in the A and B channels is stronger in theory. Second, another resonance is present at 38 eV in the calculations but is not observed experimentally.

C. Ellipticity dependence of the harmonic emission

The transverse momentum distribution of the electrons at the exit of the tunnel is dictated by the molecular orbital structure, filtered through the tunneling process [7]. If the electron wavepacket at the exit of the tunnel is spatially localized, then the momentum distribution is broad. The transverse momentum distributions associated with argon and the A and C channels in SF_6, calculated using the approach described in [7], are shown in Supplementary Figure Supplementary Figure 4. Calculations for the X and B channels would require a better modelling because of the presence of multiple nodal planes. The distributions have a Gaussian profile, with the same width for argon and the A channel, while the C channel shows a broader distribution. This is consistent with the more localized character of the HOMO-3 (C) rather than the diffuse HOMO-1 (A). During propagation in the continuum, the electron wavepacket spreads transversly. The spread of the returning electron wavepacket depends on the time spent in the continuum (and thus on the harmonic order, the lowest harmonics being emitted before the highest harmonics, but also on the initial transverse momentum distribution: the broader the initial transverse momentum distribution, the broader the returning wavepacket. With an elliptically polarized driving field, the orthogonal component of the field shifts the electron in the lateral direction, leading to a suppression of the recollision probability. Such suppression depends on both the trajectory length and the initial channel-specific transverse momentum. Therefore, the harmonic response β as a function of ellipticity can serve as a fingerprint of the molecular orbital, i.e. of the SFI channel.

The variation of β_{SF_6}/β_{Ar} with harmonic order is shown in Supplementary Figure Supplementary Figure 4(b). Normalization to the response of argon suppresses the linear dependence of the ellipticity response with harmonic order, which simply reflects the change in electron trajectory length. Above H17, the ellipticity responses of SF_6 and argon are remarkably similar, while they differ by $\sim 20\%$ for H15 and below. Theoretical ellipticity responses are calculated using Gaussian fits of the distributions shown in Supplementary Figure Supplementary Figure 4(a) and reported, in Supplementary Figure Supplementary Figure 4(b), upon normalization to the argon response. There is a very good agreement between the argon response and that of the A channel above H17. The lower part of the spectrum shows a clear discrepancy, which could either be the signature of a channel switching, or of strong recombination effects related to a resonance. The analiysis of the harmonic polarization state (Fig. 5 of the main text) lead us to conclude that the resonance is responisble for the deviation.

III. SUPPLEMENTARY REFERENCES

[1] Stanton, J. F., Gauss, J. *et al.* CFOUR, Coupled-Cluster techniques for Computational Chemistry, a quantum-chemical program package. *http://www.cfour.de.*

[2] Schmidt, M. W. *et al.* General Atomic and Molecular Electronic Structure System. *Journ. Comput. Chem.* **14**, 1347 (1993).

[3] Holland, D.M.P. & MacDonald, M.A. & Baltzer, P. & Karlsson, L. & Lundqvist M. & Wannberg, B. & von Niessen, W. , An experimental and theoretical study of the valence shell photoelectron spectrum of sulphur hexafluoride. *Chemical Physics* **192**, 333 (1995).

[4] Yang, L. *et al.* Energy-dependent valence photoelectron spectra of SF6. ab initio calculations and measurements. *Journal of Electron Spectroscopy and Related Phenomena* **94**, 163–179 (1998).

[5] Jose, J. & Lucchese, R.R. & Rescigno, T.N. , Interchannel coupling effects in the valence photoionization of SF6. *The Journal of Chemical Physics* **140**, 204305 (2014).

[6] Stener, M., Toffoli, D., Fronzoni, G. & Decleva, P. Time dependent density functional study of the photoionization dynamics of SF6. *The Journal of Chemical Physics* **124**, 114306–114306–13 (2006).

[7] Murray, R., Spanner, M., Patchkovskii, S. & Ivanov, M. Tunnel ionization of molecules and orbital imaging. *Physical Review Letters* **106**, 173001 (2011).
